# Importance of systemic redox homeostasis biomarkers and transcription factors in patients undergoing open-heart surgery with cardiopulmonary bypass

**DOI:** 10.1007/s00595-025-03026-w

**Published:** 2025-03-18

**Authors:** Jamila Tahmazli, Şeydanur Turgut, Tamer Cebe, Fatih Kızılyel, Erdem Atasever, Ayhan Üğüden, Bülend Ketenci, Gülnur Andican, Ufuk Çakatay

**Affiliations:** 1 Department of Medical Biochemistry, Cerrahpaşa Faculty of Medicine, Istanbul University-Cerrahpaşa, Istanbul, Türkiye; 2 Department of Cardiovascular Surgery, Dr. Siyami Ersek Thoracic and Cardiovascular Surgery Training and Research Hospital, Istanbul, Türkiye

**Keywords:** Cardiopulmonary bypass, Open-heart surgery, Redox biomarkers, Redox homeostasis, Redox transcription factors

## Abstract

**Purpose:**

Patients undergoing coronary artery bypass graft surgery and isolated valve disease surgery may experience redox dyshomeostasis associated with cardiopulmonary bypass (CPB).

**Methods:**

We investigated the impact of CPB on systemic redox homeostasis by analyzing redox biomarkers and antioxidant transcription factors preoperatively and postoperatively using spectrophotometric and immunochemical methods.

**Results:**

Our findings indicate significant variations in protein oxidation biomarkers, antioxidant capacity biomarkers, and transcription coactivator peroxisome proliferator-activated receptor-gamma coactivator-1α (PGC-1α) levels after CPB. The ROC analysis indicated that protein carbonyl was valuable in the preoperative (*p* = 0.009) and postoperative (*p* = 0.013) periods. We also found that glutathione peroxidase was a valuable redox biomarker during the postoperative period (*p* = 0.000). An ROC analysis of catalase activity (*p* = 0.017) before CPB indicated the importance of catalase in eliminating increased hydroperoxide load. The ROC graphs reinforced the value of PGC-1α (*p* = 0.000) as a biomarker, showing a similar trend to that of catalase before CPB.

**Conclusion:**

The earlier view of “increased oxidative stress and decreased biofunction” has shifted to exploring the physiological role of redox signaling regulation. We believe that future studies on the effects of CPB on systemic redox regulation processes through redox signaling mechanisms will significantly contribute to the relevant literature.

## Introduction

It is known that the rate of reactive oxygen species (ROS)-mediated oxidative damage increases in myocardial tissues in cardiovascular diseases [1]. Histopathological changes observed in myocardial tissue related to cardiovascular aging occur as a consequence of macromolecular oxidative damage resulting from impaired redox homeostasis [2]. Owing to the limited regenerative capacity of postmitotic cardiomyocytes, repair capacities are also limited in response to oxidative damage. In addition, ROS are considered hormetic, effective cellular redox signals that facilitate transmission through adaptive proteins in cardiovascular tissue [3, 4]. Physiological levels of ROS regulate cell proliferation, differentiation, and excitation–contraction coupling in cardiomyocytes through redox signaling mechanisms.

Cardiovascular diseases cause damage to cardiomyocytes by disrupting hemodynamic and ischemic processes. Redox dyshomeostasis is primarily caused by reactive oxygen species (ROS) produced by inflammatory cells in the myocardial tissue or vascular system. Systemic inflammatory responses during cardiac surgery affect the redox status of plasma proteins and blood cells, leading to redox dyshomeostasis. The imbalance in homeostatic regulation between ROS formation and neutralization results in the excessive accumulation of oxidation products, contributing to cardiovascular diseases such as hypertension, atherosclerosis, diabetic vascular disease, myocardial ischemia–reperfusion injury, and heart failure [4, 5]. Both cardiovascular disease and cardiopulmonary bypass (CPB) procedures are associated with impaired redox homeostasis, potentially resulting in ROS-mediated tissue and organ damage after cardiac surgery [6]. ROS signaling has essential physiological consequences for vascular repair. In addition to response to local stimuli (growth factors, hormones, and cytokines), they also have the task of starting many cellular processes, including adhesion, angiogenesis, cell migration, contraction, and proliferation. Redox signaling pathways are disrupted in chronic vascular diseases by the gradual accumulation of oxidative damage in macromolecules such as proteins, lipids, and DNA. ROS also activate transcription factors, such as Nrf2, in vascular cells. Unregulated apoptosis and cellular aging are caused by increased ROS production, which results in insufficient and delayed repair processes. Advanced aging causes increased vascular insufficiency, resulting in insufficient tissue perfusion [7, 8].

Transcription factors, such as nuclear factor erythroid 2-related factor 2 (Nrf2) and peroxisome proliferator-activated receptor-gamma coactivator-1α (PGC-1α), play important roles in controlling physiological functions through redox regulation mechanisms in myocardial tissue. Nrf2 is a crucial transcription factor that stimulates the expression of enzymatic antioxidant systems, transcription factors, redox signaling proteins, protein cofactors, and cytoprotective detoxifying enzymes in response to oxidative stress during the pathogenesis of cardiovascular diseases. It also plays a protective role against oxidative stress during CPB. The Nrf2 transcription factor induces the activation of structural genes encoding enzymes such as NAD(P)H dehydrogenase (quinone 1) (NQO1), cytosolic and mitochondrial/manganese superoxide dismutase (MnSOD), catalase (CAT), and glutathione peroxidases (GPx) in redox signaling pathways through the ARE regulator nucleotide sequence [9]. Furthermore, Nrf2-Keap1 (Kelch-like ECH-associated protein 1) is critical for controlling redox homeostasis in cardiac tissues [10, 11]. Nrf2 is a transcription factor that is sensitive to changes in redox status that interacts with Keap1, a protein that plays a role in the regulation of redox homeostasis in myocardial tissue. PGC-1α, in contrast, acts as a coactivator that activates transcription factors in different metabolic pathways. It promotes mitochondrial biogenesis, oxidative metabolism, neovascularization, and fatty acid oxidation in heart muscle [12]. PGC-1α controls the expression of antioxidant genes associated with Nrf2 activation [13].

Under physiological conditions, the rate of ROS formation and antioxidant defense capacity remain in a homeostatic balance. High levels of ROS or reduced antioxidant defense capacity lead to structural and functional changes in biomolecules, causing oxidative damage. During cardiac surgery, ischemia- and reperfusion-induced myocardial damage occur in all patients [14]. Non-biological surfaces have both direct and indirect effects on blood components. Contact with non-endothelial surfaces during CPB and valve surgery can lead to blood cell damage. The main causes of oxidative stress include non-pulsatile flow, contact of blood with non-endothelial surfaces, cross-clamping of heart blood flow, anesthetic drugs, myocardial damage, the complement system, and reperfusion. On the other hand, neutrophils, catecholamines, the complement system, cytokines released from activated neutrophils, endothelial damage, the kallikrein cascade, and endotoxin release play a role in the development of sterile inflammatory reactions [15]. Ischemia and reperfusion injury can be observed in myocardial tissues of patients undergoing coronary artery bypass graft surgery and isolated valve replacement surgery [14, 16, 17]. Comparative research examining the effects of these redox variations on systemic redox homeostasis is not currently available in the relevant literature. In this study, we aimed to contribute to the development of effective strategies to minimize oxidative damage associated with cardiac surgery by investigating the effects of coronary artery bypass graft surgery on systemic redox homeostasis during the preoperative and postoperative periods using redox biomarkers and antioxidant system transcription factors. We also plan to compare these redox effects with those observed in elective isolated valve replacement surgery in patients without coronary artery disease. These patients share a similar pathogenesis, and we aim to identify potential strategies to minimize oxidative damage associated with cardiac surgery using CPB.

## Methods

### Study design and participants

The study included 384 patients who underwent coronary artery bypass grafting (CABG), 96 patients who had mitral valve interventions, and 135 patients scheduled for aortic valve replacements during open-heart surgery between January 2023 and July 2023. Among these, 54 patients were prospectively selected based on the eligibility criteria and analyzed along with their outcomes. Patients included in our study were selected from among those who underwent elective coronary artery bypass grafting (*n* = 28) and those who underwent elective isolated valve replacement surgery without coronary artery disease (*n* = 26 [aortic valve, *n* = 23; mitral valve, *n* = 3]) at Dr. Siyami Ersek Chest, Heart, and Vascular Surgery Training and Research Hospital between January 2023 and July 2023. The study sample size was calculated to be a minimum of 24 people, using a 95% confidence interval and a power ratio of 0.95. The analysis was performed using the G-Power statistical analysis program (ver. 3.1.9).

**Fig. 1:**
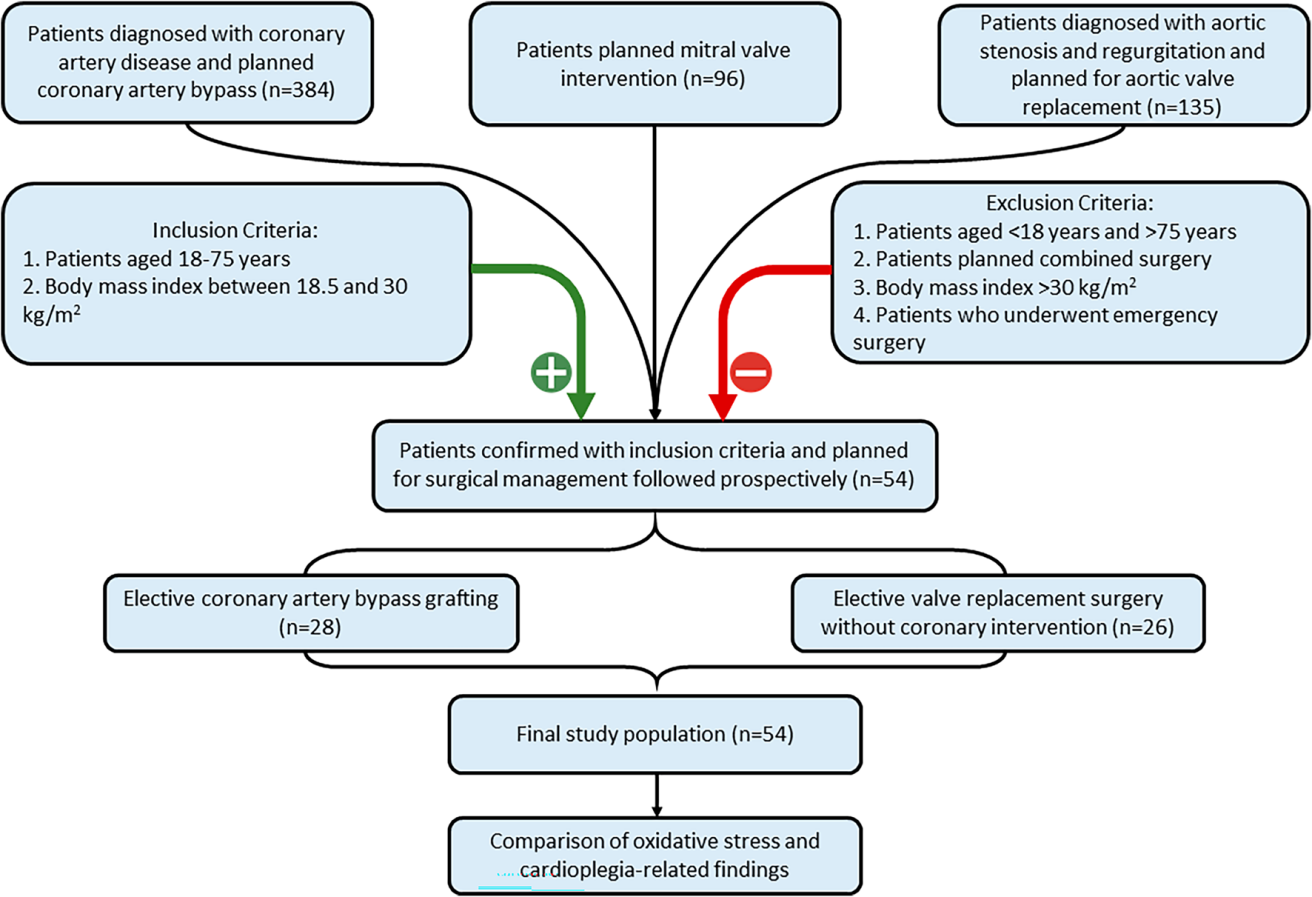
Patient selection flow chart

The following inclusion criteria were applied: 1) age 18–75 years; elective coronary artery bypass surgery for coronary artery disease; absence of coronary artery disease treated by elective valve replacement surgery; body mass index 18.5–30 kg/m^2^. The following exclusion criteria were applied: age < 18 years or > 75 years; patients requiring combined coronary artery bypass and valve surgery; obese patients (body mass index > 30 kg/m^2^); patients undergoing emergency coronary bypass surgery (Fig. 1).

### Patient data

Data for 28 patients who underwent coronary artery bypass graft surgery and 26 patients who underwent isolated valve surgery are presented in Table 1. We compared the general characteristics of these two patient groups, both of which experienced oxidation-related degenerative processes. The study focused on comparing these groups to a control group of individuals with similar demographic and preoperative characteristics, where no statistically significant differences were observed. This study focused on comparing coronary artery bypass graft surgery patients to isolated valve surgery control cases with similar demographic and preoperative characteristics, where no statistically significant differences were observed The insignificant differences in demographic/laboratory values in Table 1 indicate that our cardiopulmonary bypass-related oxidation findings are independent of conditions such as age, sex, and preoperative and routine biochemistry data. Our aim was to focus on coronary bypass and valve patients in the patient and control groups. Cardiovascular diseases related to atherosclerosis are not limited to the coronary arteries; they affect all arteries systemically. Therefore, to investigate the systemic effects of cardiovascular disease during cardiopulmonary bypass, we compared a patient group with valvular disease who did not have documented atherosclerotic cardiovascular disease.

**Table 1:** Patient data

	Coronary patient group (*n* = 28)	Valve patient group (*n* = 26)	*p*
*Sex*			
Male	24 (85.7%)	18 (69.2%)	0.146
Female	4 (14.3%)	8 (30.8%)	0.275
Age	61.43 ± 6.292	58.15 ± 11.712	0.275
BMI	27.38 ± 4.18	26.87 ± 5.05	0.627
*DM*			
Insulin	7 (25%)	2 (7.7%)	0.093
OAD	8 (28.6%)	3 (11.5%)	
None	13 (46.4%)	21 (20.8%)	
Hypertension	24 (85.7%)	20 (76.9%)	0.103
Hyperlipidemia	19 (67.9%)	9 (34.6%)	0.142
CKD	5 (17.9%)	5 (19.2%)	0.065
COPD	5 (17.9%)	4 (15.4%)	0.633
Smoking	21 (75%)	14 (53.8%)	0.083
Total cholesterol (mg/dL)	191.14 ± 44.51	192.00 ± 36.58	0.910
High-density lipoprotein cholesterol (HDLc) (mg/dL)	37.36 ± 7.631	39.96 ± 6.384	0.165
Low-density lipoprotein cholesterol (LDLc) (mg/dL)	119.32 ± 40.35	109.12 ± 35.64	0.416
C-reactive protein (CRP) (mg/L)	94.68 ± 25.56	88.88 ± 33.40	0.153
Cross-clamp Time	73.43 ± 19.323	84.23 ± 22.079	0.083
CPB Time	109.25 ± 24.517	119.85 ± 28.335	0.185
Preop EF%	53.21 ± 8.630	54.23 ± 7.442	0.676
Weaning CPB time	35.82 ± 13.955	35.62 ± 12.949	0.910

*p* > 0.05 for all parameters

### Surgical procedure

All patients underwent full sternotomy, and cardiopulmonary bypass was performed under mild hypothermia at 32 °C for cardioprotection. The initial dose of cardioplegia solution (4 parts blood, 1 part crystalloid) was administered in an antegrade manner with a volume of 1000 mL. This was followed by continuous administration or doses given every 15–20 min in both retrograde and antegrade fashion. A simultaneous focal ice slash was applied to promote hypothermia. After distal coronary anastomoses, proximal anastomoses were performed for aortic cross-clamping. Cardioplegic solution was administered as a standard antegrade in valve patients without coronary artery disease (aortic valve, *n* = 23; mitral valve, *n* = 3). Cardioplegia was initially administered at a dose of 1000 mL, followed by continuous administration or doses given every 15–20 min in both retrograde and antegrade fashion.

Following the successful completion of the surgical procedure, the aortic cross-clamp was removed. After providing adequate support for one-third of the cross-clamp duration, cardiopulmonary bypass was terminated once the temperature and pressure returned to normal.

### Sample collection and storage

To investigate the effects of CPB on the included patients, venous blood samples were collected from systemic circulation into plain tubes containing yellow caps half an hour before CPB and within the first half hour after CPB. Plain tubes without additive-containing venous blood samples were centrifuged at 3000 rpm for 15 min at 4 °C to obtain serum samples. To analyze redox biomarkers and transcription factors, serum aliquots were frozen and stored at − 80 °C until use.

### Analysis of redox biomarkers and related signaling pathway factors

Reversing or preventing systemic oxidative damage in plasma constituents, such as proteins, lipids, and redox-related factors is the primary goal of developing effective therapeutic strategies to prevent or treat age-related vascular disorders. Redox biomarkers of plasma components were classified into four major categories, as previously described by Cebe et al. [2]. In this study, we investigated the effects of CPB on systemic redox homeostasis by examining redox biomarkers and antioxidant system transcription factors in the preoperative and postoperative periods using spectrophotometric and immunochemical methods (Table 2).

**Table 2:** Assayed redox biomarkers and related signaling pathway activation factors

Biomarker groups	Parameter	Feature	Analytic method
Protein oxidation biomarkers	PCO	Early and stable protein oxidation biomarker [18]	Colorimetric manual [19] CV% = 3.2
	AOPP	Carried by albumin which is the most important antioxidant molecule of plasma [20]	Colorimetric manual [20] CV% = 3.0
	P-SH	Specific reductants of individual protein disulfate bonds (thioredoxin) [21]	Colorimetric manual [22, 23] CV% = 1.8
Lipid peroxidation biomarker	LOOH	Participate in redox reactions often determining magnitude of lipid peroxidation [24]	Colorimetric manual [25, 26] CV% = 2.4
Antioxidant capacity biomarkers	MnSOD level	Expressed enzyme protein level	ELISA CV% < 4
	GPx activity	Enzyme which is responsible for the removal of lipid hydroperoxides and H_2_O_2_ [24]	Colorimetric kit BT-LAB (Shanghai Korain Biotech Co, Shanghai, China) CV% < 5.7
	GPx level	Expressed enzyme protein level	ELISA BT-LAB (Shanghai Korain Biotech Co, Shanghai, China) CV% < 4.17
	CAT activity	Enzyme which is responsible for the decomposition of H_2_O_2_ [27]	Spectrophotometric manual [27] CV% = 2.8
	CAT level	Expressed enzyme protein level	ELISA (Shanghai Korain Biotech Co, Shanghai, China) CV% < 10
	Np-SH	As a redox buffer in plasma, Np-SH groups include thiol groups of homocysteine, glutathione, and coenzyme A [21]	Colorimetric manual [22] CV% = 1.2
	T-SH	The sum of protein and non-protein thiol groups in plasma [21]	Colorimetric manual [22]. CV% = 1.4
Transcription factors	Nrf2	Nrf2 is a transcription factor that stimulates the expression of enzymatic antioxidant systems, transcription factors, redox signaling proteins, protein cofactors, and cytoprotective detoxifying enzymes [28]	ELISA (Shanghai Korain Biotech Co, Shanghai, China) CV % < 10
	Keap1	Keap1 interact with Nrf2 to regulate antioxidant response [29]	ELISA (Shanghai Korain Biotech Co, Shanghai, China) CV % < 8
	PGC-1α	PGC-1α regulates physiological functions of myocardial tissue through redox regulation mechanisms [28]	ELISA (Shanghai Korain Biotech Co, Shanghai, China) CV% < 8

*PCO*, protein carbonyl groups; *AOPP*, advanced oxidation protein products; *P-SH*, protein thiol groups; *LOOH*, lipid hydroperoxide groups; *MnSOD*, mitochondrial superoxide dismutase; *GPx*, glutathione peroxidase; *CAT*, catalase; *Np-SH*, non-protein thiol groups; *T-SH*, total thiol groups; *Nrf2*, nuclear factor erythroid 2-related factor 2; *Keap1*, Kelch-like ECH-associated protein 1; *PGC-1α*, peroxisome proliferator-activated receptor-gamma coactivator-1 alpha

### Ethical statement

The study protocol was approved by the local Ethics Committee of the Cerrahpasa Faculty of Medicine, Istanbul University-Cerrahpasa (File number: 307, Date: 06/09/2022) and was conducted in accordance with the Declaration of Helsinki.

### Statistical analyses

The sample size for comparing the means of the two groups was calculated using G-Power (ver. 3.1.9). The statistical analysis of the data was conducted using SPSS (ver. 29.0). Descriptive statistics in our study are expressed as the mean ± standard deviation (mean ± SD). The normality of the data distribution was assessed using the Kolmogorov–Smirnov test. Non-normally distributed data for parametric variables were analyzed using the non-parametric Mann–Whitney U test and Wilcoxon signed test, while normally distributed data were analyzed using the parametric independent-samples *t* test. Correlation analyses were performed using Pearson’s correlation coefficient for normally distributed data and Spearman’s correlation coefficient for non-normally distributed data. *p* values of < 0.05 were considered to indicate statistical significance, with a confidence level of 95%. A receiver operating characteristic (ROC) curve analysis was used to examine the sensitivity and specificity of redox biomarkers.

## Results

### Redox and antioxidant system biomarker-related findings

The mean ± SD levels of redox biomarkers in venous blood samples collected half an hour before and half an hour after CPB surgery in 28 patients and isolated valve surgery in 26 patients are presented in Table 3.

**Table 3:** Redox biomarker and transcription factor-related findings

Biomarker groups	Parameter	Pre-CPB venous blood		Post-CPB venous blood	
		Coronary patient group (*n* = 28)	Valve patient group (*n* = 26)	Coronary patient group (*n* = 28)	Valve patient group (*n* = 26)
Protein oxidation biomarkers	PCO (nmol/mg protein)	**2.24 ± 0.65**^**a,b**^	**2.46 ± 0.96**^**b**^	**1.93 ± 0.26**^**a**^	2.12 ± 0.56
	AOPP (µmol chloramine-T equivalent /L)	36.46 ± 6.94	36.1 ± 6.70	36.94 ± 5.49	37.41 ± 7.25
	P-SH (mmol/L)	0.12 ± 0.09	**0.08 ± 0.07**^**b**^	**0.12 ± 0.09**^**a**^	0.19 ± 0.14
Lipid peroxidation biomarker	LOOH (µmol/L)	0.54 ± 0.21	0.61 ± 0.18	0.49 ± 0.17	0.55 ± 0.19
Antioxidant capacity biomarkers	MnSOD levels (ng/mL)	**26.78 ± 6.92**^**a**^	22.01 ± 11.57	25.97 ± 6.51	24.44 ± 8.89
	GPx activity (U/mg protein)	0.40 ± 0.07	**0.36 ± 0.11**^**b**^	**0.39 ± 0.07**^**a**^	0.29 ± 0.09
	GPx levels (µU/mL)	139.89 ± 42.52	159.16 ± 50.02	**131.66 ± 30.77**^**a**^	162.03 ± 31.98
	CAT activity (kU/mg protein)	**276,562.99 ± 315,148.68**^**a**^	377,846.01 ± 287,072	264,182.64 ± 280,712.73	274,658.03 ± 148,332.42
	CAT levels (KU/L)	179.62 ± 58.68	195.59 ± 113.64	191.87 ± 62.49	196.23 ± 77.17
	Np-SH (mmol/L)	0.21 ± 0.03	0.20 ± 0.03	**0.21 ± 0.03**^**a**^	0.19 ± 0.02
	T-SH (mmol/L)	0.33 ± 0.17	**0.27 ± 0.07**^**b**^	0.33 ± 0.14	0.37 ± 0.15
Transcription factors	Nrf2 (ng/mL)	9.64 ± 2.97	**9.53 ± 2.05**^**b**^	10.44 ± 2.49	11.50 ± 2.28
	Keap1 (ng/L)	**433.86 ± 165.54**^**b**^	**469.66 ± 148.09**^**b**^	515.33 ± 128.62	529.61 ± 186.89
	PGC-1α (ng/mL)	**5.85 ± 2.04**^**a**^	**4.88 ± 2.45**^**b**^	6.17 ± 2.29	6.09 ± 3.08

*p* < 0.05 values are shown in bold

*CPB*, cardiopulmonary bypass; *PCO*, protein carbonyl groups; *AOPP*, advanced oxidation protein products; *P-SH*, protein thiol groups; *LOOH*, lipid hydroperoxide groups; *MnSOD*, mitochondrial superoxide dismutase; *GPx*, glutathione peroxidase; *CAT*, catalase; *Np-SH*, non-protein thiol groups; *T-SH*, total thiol groups; *Nrf2*, nuclear factor erythroid 2-related factor 2; *Keap1*, Kelch-like ECH-associated protein 1; *PGC-1α*, peroxisome proliferator-activated receptor-gamma coactivator-1 alpha

^a^vs. valve patient group, ^b^vs. post-CPB venous blood

### Protein oxidation-related findings

PCO (nmol/mg protein), a significant decrease in PCO levels was observed in venous blood samples collected before (2.24 ± 0.65) and after (1.93 ± 0.26) CPB surgery in the coronary artery patient group (*p* = 0.016). In the valve patient group, a significant decrease was observed in venous blood samples collected before (2.46 ± 0.96) and after (2.12 ± 0.56) CPB surgery (*p* = 0.022). Pre-CPB venous blood samples showed that PCO levels were significantly lower in the coronary patient group than in the valve patient group (*p* = 0.012). In post-CPB venous blood samples, the PCO levels were significantly lower in the coronary patient group than in the valve patient group (*p* = 0.019). AOPP (µmol chloramine-T equivalent/L). No significant differences were found between groups in the pre-CPB or post-CPB venous blood samples. In post-CPB venous blood samples P-SH levels were significantly lower in the coronary patient group (0.12 ± 0.09 mmol/L) than in the valve patient group (0.19 ± 0.14 mmol/L) (*p* = 0.035) (Table 3).

### Lipid oxidation-related findings

No significant differences in LOOH (µmol/L) were found between the groups in venous blood samples collected before and after CPB surgery (Table 3).

### Antioxidant capacity biomarkers-related findings

MnSOD levels (ng/mL) in venous blood samples before CPB surgery were significantly higher in the coronary artery patient group than in the valve patient group (*p* = 0.035). In post-CPB venous blood samples, GPx activity (U/mg protein) was significantly higher in the coronary patient group than in the valve patient group (*p* < 0.001). In post-CPB venous blood samples, GPx levels (µU/mL) were significantly lower in the coronary patient group than in the valve patient group (*p* = 0.001). In pre-CPB venous blood samples, CAT activity (kU/mg protein) was significantly lower in the coronary patient group than in the valve patient group (*p* = 0.023). No significant differences in CAT levels (kU/L) were found between the groups in venous blood samples collected before and after CPB surgery. In post-CPB venous blood samples, the levels of Np-SH (mmol/L), which mainly include the reduced form of glutathione (GSH), were significantly higher in the coronary patient group than in the valve patient group (*p* = 0.010). No significant differences in T-SH (mmol/L) were found between the groups in venous blood samples collected before and after CPB surgery (Table 3).

### Transcription factors-related findings

No significant differences were found between the groups in Nrf2 (ng/mL) levels in venous blood samples collected before and after CPB. No significant differences were found between the groups in Keap1 (ng/L) levels in venous blood samples collected before and after CPB surgery. PGC-1α (ng/mL) levels in pre-CPB venous blood samples were significantly increased in the coronary patient group in comparison to the valve patient group (*p* = 0.035) (Table 3).

### Correlations

The results of correlation analysis results among various redox biomarkers, transcription factors, and CRP levels in venous blood samples collected before and after CPB are shown in (Tables 4 and 5).

**Table 4:** Correlation analysis in the coronary patient group

		Post-CPB PCO	Post-CPB AOPP	Post-CPB P-SH	Pre-CPB LOOH	Pre-CPB GPx Activity	Pre-CPB CAT Levels	Post-CPB CAT Levels	Pre-CPB MnSOD Levels	Post-CPB MnSOD Levels	Pre-CPB Nrf2	Post-CPB Nrf2	Pre-CPB Keap1	Post-CPB Keap1	Pre-CPB PGC-1⍺	Post-CPB PGC-1⍺
Pre-CPB PCO	r *p* value	.714** 0.000	–	–	–	.385* 0.043	–	–	–	–	–	–	–	–	–.415* 0.028	–
Pre-CPB GPx Activity	r *p* value	–	–	− .390* 0.040	.558** 0.002	–	–	–	–	–	–	–	–	–	–	–
Pre-CPB GPx Levels	r *p* value	–	–	–	–	–	.579** 0.001	–	.573** 0.001	.563** 0.002	.478* 0.010	.465* 0.013	.568** 0.002	.530** 0.004	.735** 0.000	.651** 0.000
Post-CPB GPx Levels	r *p* value	–	–	–	–	–	–	.473* 0.011	.422* 0.025	–	.510** 0.006	–	–	.557** 0.002	.412* 0.029	.380* 0.046
Pre-CPB PGC-1⍺	r *p* value	–	–	–	–	–	.579** 0.001	–	.469* 0.012	–	.472* 0.011	–	.528** 0.004	–	–	.745** 0.000
CRP	r *p* value	–	.433* 0.021	–	–	–	–	–	–	–	–	–	–	–	–	–

**Table 5:** Correlation analysis in the valve patient group

		Pre-CPB PCO	Post-CPB PCO	Post-CPB AOPP	Pre-CPB P-SH	Pre-CPB LOOH	Post-CPB LOOH	Pre-CPB GPx Levels	Pre-CPB CAT Levels	Post-CPB CAT Levels	Pre-CPB MnSOD Levels	Post-CPB MnSOD Levels	Pre-CPB Nrf2	Post-CPB Nrf2	Pre-CPB Keap1	Post-CPB Keap1
Pre-CPB PCO	r *p* value	–	.718** 0.000	–	–	.469* 0.016	–	–	–	–	–	–	–	–	–	–
Post-CPB PCO	r *p* value	.718** 0.000	–	.569** 0.002	–	–	–	–	–	–	–	–	–	–	–	− .561** 0.003
Pre-CPB GPx activity	r *p* value	.495* 0.010	.552** 0.003	–	− .478* 0.013	–	–	–	.393* 0.047	–	.594** 0.001	.522** 0.006	–	–	− .478* 0.014	− .439* 0.025
Post-CPB GPx activity	r *p* value	–	–	–	–	–	–	–	.400* 0.043	–	.500** 0.009	–	–	–	–	–
Pre-CPB GPx levels	r *p* value	–	–	–	–	–	–	–	.481* 0.013	.540** 0.004	–	–	.536** 0.005	.720** 0.000	.544** 0.004	.442* 0.024
Post-CPB GPx levels	r *p* value	–	–	–	–	–	–	.819** 0.000	.426* 0.030	.485* 0.012	–	–	.449* 0.022	.759** 0.000	.411* 0.037	–
Pre-CPB CAT activity	r *p* value	.400* 0.043	–	.493* 0.011	–	.447* 0.022	− .543** 0.004	–	–	–	–	− .423* 0.031	–	–	–	–
Post-CPB CAT activity	r *p* value	–	–	–	–	–	–	.406* 0.039	.571** 0.002	–	–	–	.427* 0.030	–	.393* 0.047	–
Post-CPB Np-SH	r *p* value	–	− .517** 0.007	− .394* 0.047	–	–	–	–	–	–	–	–	–	–	–	–
CRP	r *p* value	–	–	–	–	–	–	–	–	− .435* 0.026	− .403* 0.041	− .485* 0.012	− .424* 0.031	− .522** 0.006	–	–

### ROC analysis

#### ROC analysis of oxidative system biomarkers

**Fig. 2:**
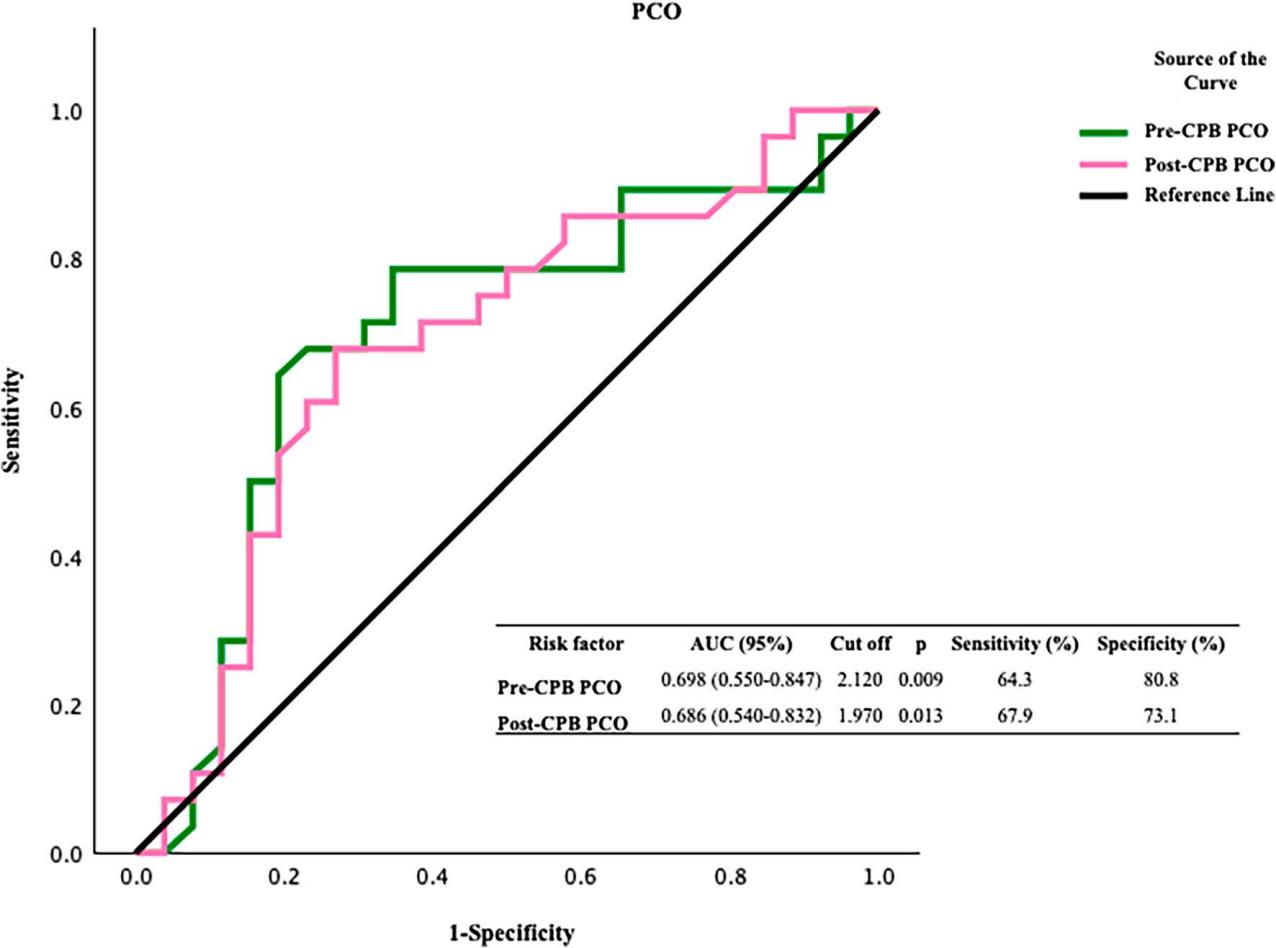
ROC curves for the protein carbonyl (PCO) groups

The ROC analysis of oxidant system biomarkers indicated that PCO groups were valuable redox biomarkers before and after CPB, highlighting the diagnostic value of the PCO group in evaluating the impact of CPB on the oxidant load in systemic circulation (Fig. 2).

#### ROC analysis of antioxidant system biomarkers

**Fig. 3:**
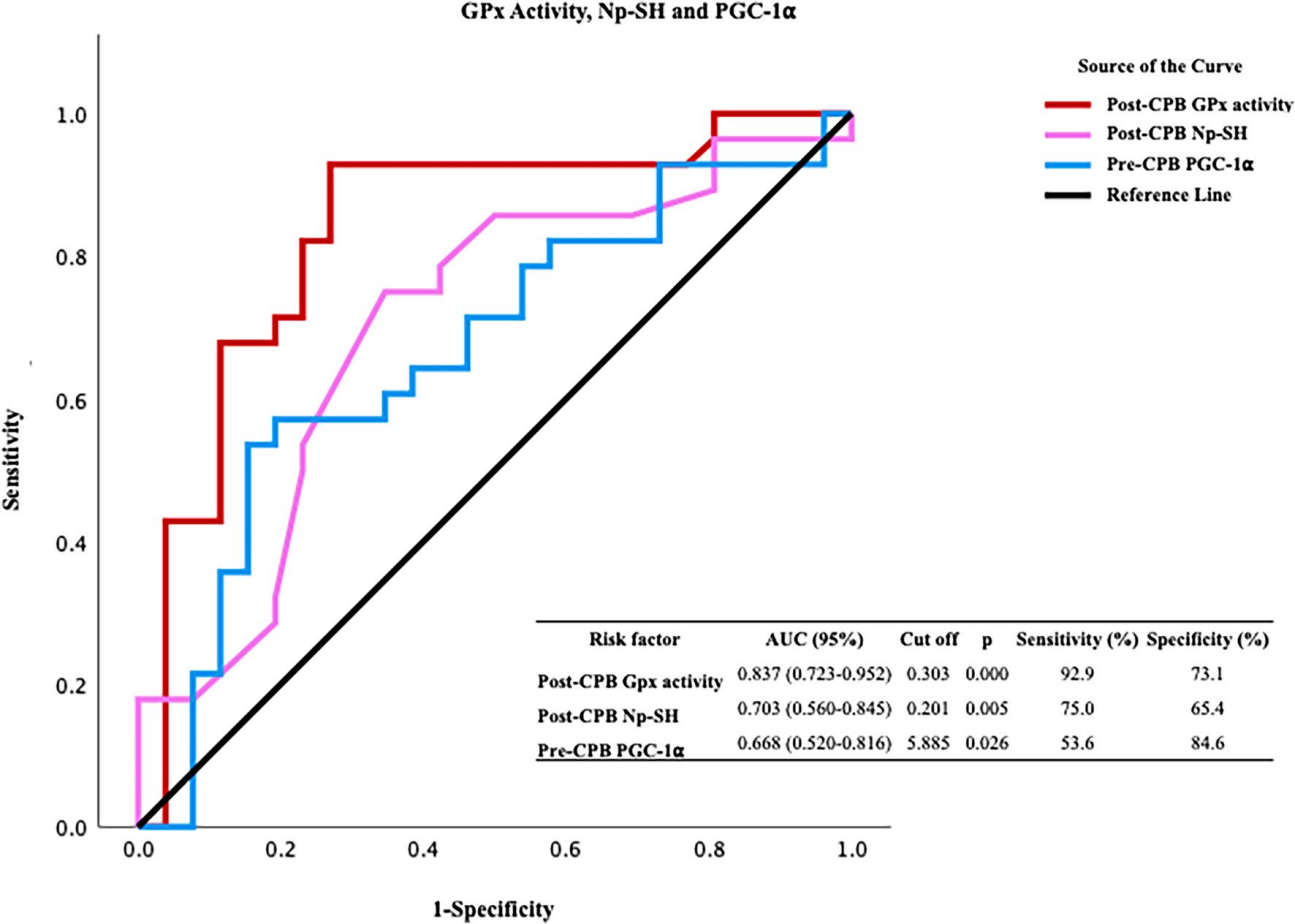
ROC curves for glutathione peroxidase (GPx) activity, non-protein thiol (Np-SH) groups, and peroxisome proliferator-activated receptor-gamma coactivator-1 alpha (PGC-1α) levels

**Fig. 4:**
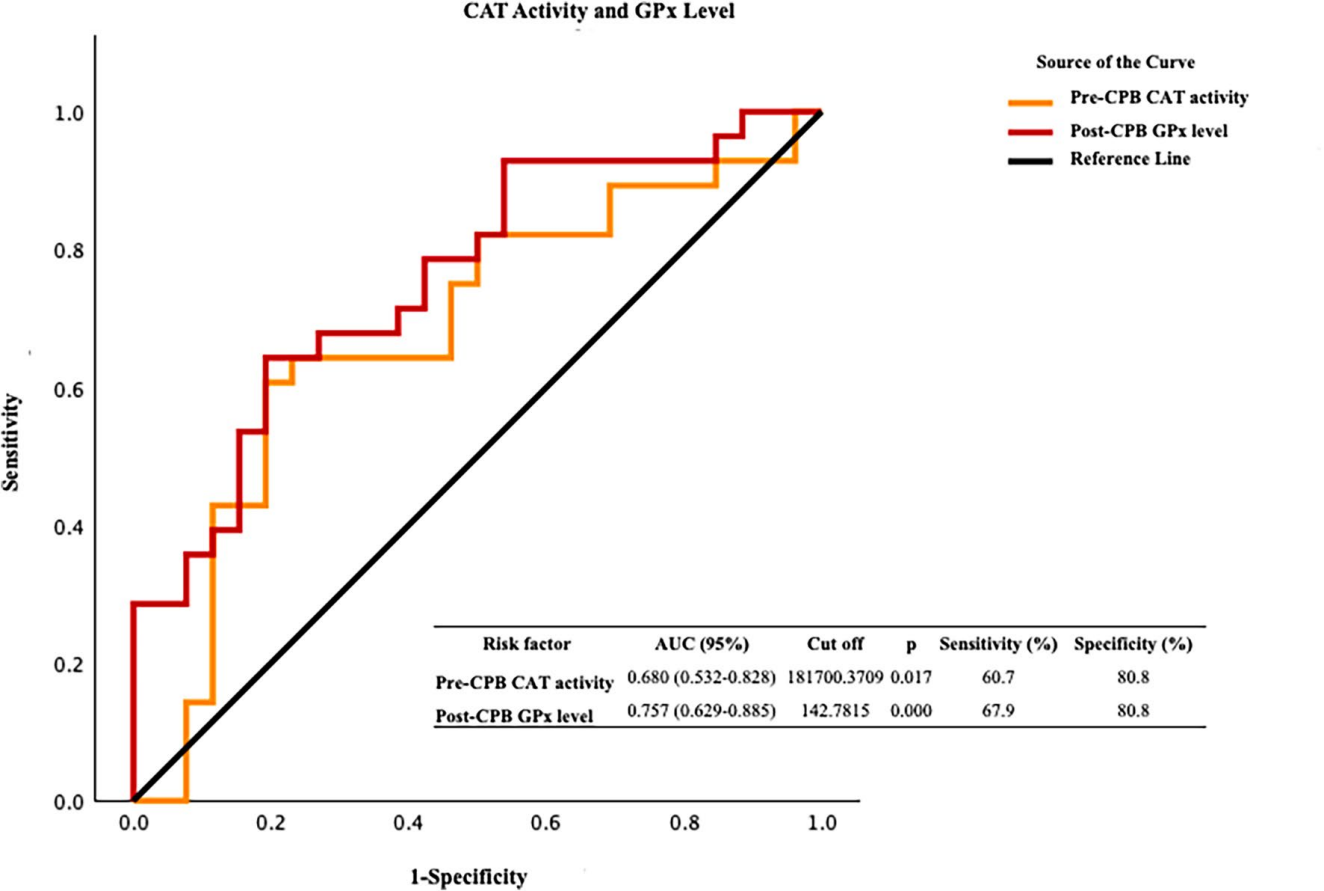
ROC curves for glutathione peroxidase (GPx) expression levels and catalase (CAT) activity

When examining the antioxidant aspect of systemic redox homeostasis, we found that GPx after CPB, in terms of both activity and expression levels, is a valuable redox biomarker. Among the changes in antioxidant biomarkers after CPB, GPx activity emerged as a highly effective diagnostic indicator. This high diagnostic performance was confirmed through ROC analysis of the PCO groups. The Np-SH fraction largely consisted of reduced glutathione and showed high performance postoperatively after CPB, suggesting a potential relationship between the substrate role of existing glutathione and its utilization by GPx (Fig. 3). The ROC analysis of CAT activity before CPB indicated the importance of CAT as an antioxidant enzyme in eliminating the increased hydroperoxide load in the systemic circulation. CAT is an enzyme whose expression or activity is induced by PGC-1α through different metabolic pathways in tissues of the cardiovascular system. The ROC graphs reinforced the value of PGC-1α as a biomarker, showing a similar trend to that of CAT before CPB (Fig. 4).

### Routine biochemical parameters

The mean ± SD values of routine biochemical parameters in venous blood samples obtained after CPB from 28 patients undergoing coronary artery bypass surgery and 26 patients undergoing isolated valve surgery are shown in Table 1. No significant differences were observed between the patient groups.

## Discussion

ROS and oxidized LDL (OxLDL) promote the infiltration of T lymphocytes and macrophages into the valve subendothelial tissue through a similar mechanism to the atherogenesis process. OxLDL promotes the formation of foam cells from monocytes in calcification-associated aortic valve diseases. The relationship between atherogenesis and redox metabolism is considered an irreversible oxidation process that occurs through different mechanisms in plasma proteins and lipoproteins and is influenced by disruptions in redox signaling mechanisms that affect cardiomyocytes and vascular cells [30–34]. Common risk factors, including reactive oxygen species (ROS), which contribute to endothelial damage, inflammation, hyperlipidemia, and oxLDL, play a crucial role in the atherogenesis associated with calcific aortic valve disease. Valves are made up of three layers, the outermost of which is composed of endothelial cells that create an extracellular matrix. The valve’s extracellular matrix contains collagen, proteoglycans, and elastin, providing mechanical support and durability. Revisions in the extracellular matrix structure caused by genetic, acquired, and environmental factors can hinder communication between endothelial and interstitial cells, resulting in valve disease. Valve surgery accounts for approximately 10–20% of all cardiovascular surgery cases [35–37]. Physiological changes in the functions of Notch homolog 1 (NOTCH1), WNT/β-catenin, and RANK occur in valve interstitial cells within the subendothelial layer, regulated by ROS, and contribute to pathological changes in the aortic valve. Activation of T cells and macrophages leads to the differentiation of valve interstitial cells in the extracellular space into myoblastic or osteoblastic cells, the accumulation of apoptotic bodies and calcified microbodies, and fibrosis [37–39].

The direct measurement of ROS and RNS formation represents a redox biomarker, although it is not easily applicable in large cohort studies as the assays need to be applied to freshly harvested tissue or biological fluid samples. Redox profiling of plasma constituents represents an emerging technique for obtaining a complete landscape of redox changes that characterize cardiovascular disease [8]. In our study, we statistically evaluated the levels of commonly used protein oxidation biomarkers (PCO, AOPP, and P-SH) in venous blood samples collected before and after CPB. PCO levels in the valve patient group were higher than those in the coronary patient group, while AOPP levels showed no significant difference between the two groups. AOPPs are reported to trigger inflammatory mechanisms, including cytokines (IL-6, IL-1), including macrophages, T lymphocytes, and mast cells, initiating the atherosclerosis process and serving as an early biomarker for atherogenesis [30–32]. In the coronary patient group, unlike in valve patients, the positive correlation between AOPP and CRP in the postoperative period suggests that AOPP may be more appropriately considered as an inflammatory marker in this group. Albumin, a major plasma protein, acts as an effective antioxidant redox buffer, eliminating hypochlorite and other free radicals involved in AOPP formation through the thiol (-SH) groups [40]. The notable reduction of P-SH levels in the postoperative CPB venous blood of the coronary patient group (relative to the valve patients) may indicate a redox regulatory mechanism that maintains lower PCO levels and prevents further increases in AOPP levels. AOPPs are new protein biomarkers of oxidative damage and represent a novel class of inflammatory mediators. AOPPs are independent risk factors for coronary artery disease [41].

GPx and CAT form a crucial antioxidant defense system against reactive LOOH in all vascular tissues and the vascular lumen. When statistically evaluating the levels of LOOH in both patient groups, a decrease in the mean values was observed due to the effect of CPB; however, this decrease was not statistically significant. The positive correlations observed in both patient groups between the activities of these enzymes and LOOH levels indicate that as LOOH levels increase, the activities of these enzymes also rise; however, they remain insufficient to significantly reduce LOOH levels.

MnSOD, the mitochondrial isoform of superoxide, is responsible for the dismutation of mitochondria-derived superoxide radicals to H_2_O_2_ [3]. When comparing the pre-CPB venous blood samples of the coronary patient group to the pre-CPB MnSOD values of the valve patient group, we observed a significant increase. This suggests that the heightened expression of MnSOD parallels the increased formation of mitochondrial superoxide radicals in the myocardial tissue of the coronary patient group. The positive correlation observed between MnSOD and Nrf2 levels in pre-CPB venous blood samples suggests that Nrf2 enhances the expression of MnSOD. The rise in GPx activity in the coronary patient group compared to the valve patient group after CPB is attributed to the elevated hydroperoxide levels. The positive correlation between GPx and CAT levels in postoperative venous blood samples from the coronary patient group suggests that both enzymes may have worked synergistically to eliminate organic hydroperoxides during the postoperative period. The rise in CAT activity noted in the pre-CPB venous blood samples of the coronary patient group highlights the role of CAT in removing the hydroperoxides generated by MnSOD. The notable rise in post-CPB Np-SH levels in the coronary patient group, compared to the valve patient group, may be due to the high content of reduced glutathione GSH).

The systemic levels of transcription factors (Nrf2 and PGC-1α) analyzed in our study were statistically evaluated using venous blood samples collected before and after CPB. PGC-1α, a coactivator that controls numerous metabolic pathways associated with ROS, plays a role in regulating the cardiovascular system, including neovascularization, mitochondrial biogenesis, oxidative metabolism, and glucose/fatty acid metabolism [12, 42]. A growing body of research suggests that dysregulation of PGC-1α is a key factor in the development and progression of HF. Relative to sham-treated animals, PGC-1α defective animals may have worse heart performance under pressure overload [43]. In our study, we found that PGC-1α levels increased in venous blood samples collected before CPB in both coronary and valve patients. This highlights the significance of PGC-1α in protecting the myocardium during CPB. Unlike PGC-1α, Nrf-2 is a transcription factor that requires pharmacological activation [44]. The transcription factor Nrf2, which protects cells from oxidative stress through natural antioxidant defense systems, is thought to be a prime candidate for therapeutic targeting in the treatment of cardiovascular diseases [45]. Natural products such as Baicalin, Anthocyanin, Diosmetin, and Hesperidin are increasingly recognized as potential Nrf2 activators with cardioprotective properties, and may thus represent a new class of therapeutic pharmaceuticals for cardiovascular diseases [33]. Keap1 inhibitors are considered to be Nrf2 activators. Recent studies indicate that Keap1 is crucial for proteostasis, mitochondrial homeostasis, cytoskeleton modulation, and cell cycle progression in the cardiovascular system, as well as for regulating Nrf2 activity. Beyond Nrf2 inactivation, the effect of these Nrf2 activators or Keap1 inhibitors on Keap1-mediated functions remains largely unclear [46]. The absence of differences in Nrf2 activation between our patient groups is due to Nrf2 dysregulation in cardiovascular disease and the lack of Nrf2 modulators in CPB. We propose that incorporating selective pharmacological agents into CPB to activate the Nrf2 signaling pathway may enhance its antioxidant effects.

The ROC analysis of oxidant systems indicated that PCO groups serve as a valuable redox biomarker, demonstrating sensitivities of approximately 64% and 68% before and after CPB, respectively, with statistically significant results. This evaluation is particularly clear at cutoff values corresponding to sensitivity levels of 0.6–0.8, indicating that the diagnostic value of PCO alone is significant for assessing the impact of CPB on systemic oxidative stress. When analyzing the antioxidant aspect of the dynamic equilibrium in systemic redox homeostasis, we found that GPx is a valuable redox biomarker after CPB, based on both its activity and expression levels. GPx activity demonstrates strong diagnostic performance with sensitivity values ranging from 0.6 to 0.9 (92.9%), which is comparable to the results of the ROC analysis for PCO groups. In contrast, the Np-SH fraction, which is made up of reduced glutathione, demonstrated high performance at cutoff values near glutathione peroxidase activity following CPB. This result indicates a connection between the use of glutathione as a substrate by GPx and elevated levels of reduced glutathione following CPB. The ROC graph of CAT activity before CPB underscores the significance of CAT as an antioxidant enzyme in reducing the elevated hydroperoxide load in systemic circulation. Among the enzymes whose expression or activity is stimulated by PGC-1α in cardiovascular tissues, the ROC curve for CAT showed similar diagnostic value to that of GPx. The ROC curve of PGC-1α, which exhibits a similar trend akin to that of CAT prior to CPB, underscores its significance as a biomarker.

## Conclusion

In the past, the widely accepted paradigm regarding ROS—increased oxidative stress, oxidative damage in macromolecules, and decreased or impaired cellular metabolic pathways function—has shifted towards investigating the physiological and pathophysiological importance of ROS-regulated redox signaling pathways [31, 47–49].

Our findings indicate significant variations in PGC-1α levels, antioxidant capacity biomarkers, and oxidized protein biomarkers after CPB. The ROC analysis of oxidant system biomarkers indicated that protein carbonyl is a valuable redox biomarker in both the preoperative and postoperative periods. We also discovered that glutathione peroxidase served as an effective redox indicator during the postoperative period. The non-protein thiol groups demonstrated high performance postoperatively during CPB. The ROC analysis of catalase activity prior to CPB highlighted the significance of catalase in reducing elevated hydroperoxide levels. The ROC graphs supported the significance of PGC-1α as a biomarker, displaying a similar pattern to that of catalase before CPB.

We believe that assessments of systemic redox status and redox signaling are necessary for routine CPB. The potential use of pharmacological agents with redoxmodulating properties, administered intravenously or via cardioplegia during cardiopulmonary bypass is expected to restore impaired redox homeostasis [50]. We believe that future studies investigating the effects of CPB on systemic redox regulation processes via redox signaling mechanisms will significantly contribute to the existing literature by assessing new redox signaling biomarkers.

## Data Availability

The original contributions presented in the study are included in the article/Supplementary Material; further inquiries can be directed to the corresponding author.

## Abbreviations

AOPP: Advanced oxidation protein products
CAT: Catalase
CPB: Cardiopulmonary bypass
GPx: Glutathione peroxidase
Keap1: Kelch-like ECH-associated protein 1
LOOH: Lipid hydroperoxide groups
MnSOD: Mitochondrial superoxide dismutase
Np-SH: Non-protein thiol groups
Nrf2: Nuclear factor erythroid 2-related factor 2
OxLDL: Oxidized LDL
PCO: Protein carbonyl groups
PGC-1α: Peroxisome proliferator-activated receptor-gamma coactivator-1 alpha
P-SH: Protein thiol groups
ROC: Receiver operating characteristic curve
ROS: Reactive oxygen species
T-SH: Total thiol groups

## Code availability

During the preparation of this study, the authors used Grammarly to improve language and readability. After using this tool/service, the authors reviewed and edited the content as required and took full responsibility for the content of the publication.
